# Enhancing Medical Education: A Pathway to Nurturing Future Healthcare Professionals

**DOI:** 10.7759/cureus.51920

**Published:** 2024-01-09

**Authors:** Mahnoor Amir, Natalie Hassan, Usama Khalid

**Affiliations:** 1 Critical Care, Basildon University Hospital, Essex, GBR; 2 Medicine, Basildon University Hospital, Essex, GBR

**Keywords:** clinical problem-solving, problem based learning (pbl), clinical learning environment, healthcare education, simulation in medical education

## Abstract

Introduction

As medical knowledge, technology, and healthcare delivery continue to evolve, it is critical that upcoming healthcare workers possess the skills and information needed to ensure optimal patient care. Numerous studies indicate that students achieve better learning outcomes through active practice rather than solely relying on theoretical knowledge. The average human attention span is only 8.25 seconds, so an effective teaching program should employ various modes and techniques to ensure that students remain involved and interested.

Aims and objectives

The aim is to identify the primary areas where medical students need teaching and guidance and form the basis of a new teaching program to meet those needs.

Materials and methods

An anonymous online questionnaire, designed by the author was distributed to medical students who came for their clinical rotations at Basildon University Hospital, Mid and South Essex NHS Foundation Trust, United Kingdom, and that laid the foundations for introducing a new teaching program at the education department of the hospital in April 2023. The progress of the teaching program was evaluated by a second questionnaire-based survey conducted after six months, in October 2023. The teaching program we designed employed various modes and techniques including simulation, flip classroom, graded quizzes, and constructive feedback. The technique we used for giving feedback to students was the “star star wish” to encourage growth and further participation. The teaching program also made use of Lev Vygotsky's “Learning zone model” to ensure optimum learning.

Results and discussion

The program received immensely positive feedback from the students, and they felt that it catered perfectly to their requirements. Twenty-three students took part in this study and the results showed that 39% of the students felt adequately prepared for ward rounds in October 2023 in contrast to only 17% in April 2023. The mean score, on a scale of 1-10 on how comfortable the students felt in discussing patient care plans and management with the rest of the team rose from 2.78 in April 2023 to 4.26 in October 2023. When asked to score how confident the students felt in performing bedside examinations in wards, 26% scored 5 or above (on a scale of 1-10) in April 2023 as compared to 62% scoring 5 or above in October 2023. The students were then asked how confident they felt in using their theoretical knowledge in practical situations and the majority scored 2 or 3 (on a scale of 1-10) in April 2023 whereas in October, the majority scored 4 or above.

Conclusions

A significant number of medical students were satisfied with the teaching program and demanded more frequent sessions. The results of this study showed that in order to foster increased student engagement and effective participation, it is essential for teaching to incorporate diverse techniques and approaches.

## Introduction

In a constantly changing healthcare environment, medical and healthcare education plays a pivotal role in shaping competent, compassionate, and skilled healthcare professionals. With advancements in medical knowledge, technological innovations, and healthcare delivery, it is imperative that future healthcare professionals are equipped with the necessary knowledge and expertise to guarantee optimum patient care. Medical education must be able to prepare future physicians for changing trends in practice patterns, the role of medicine in disease diagnosis and treatment, innovations, and advancements in medical science. This is because medicine is a rapidly evolving field that requires and can constantly incorporate newer technologies [1]. This study was conducted at the Department of Medical and Healthcare Education at Mid and South Essex, NHS Foundation Trust, United Kingdom and it investigates different methods and approaches that can be utilized to optimize the delivery of medical and healthcare education.

While active learning techniques are thought to be better than traditional lecture formats, the precise way in which these tactics should be implemented has not been as well established [2]. Because of this, curriculum designers frequently struggle to decide how to combine traditional lectures, e-learning, and small groups to provide the greatest learning environment [3,4]. The average human attention span is only 8.25 seconds [5], so a teaching program should be designed in such a way that students do not lose focus and remain engaged. This study examines several methods that could be applied to enhance students' ability to concentrate.

## Materials and methods

This cross-sectional survey-based study was conducted from April 2023 to October 2023, for six months at the Department of Medical and Healthcare Education at Basildon University Hospital, Mid and South Essex NHS Foundation Trust, United Kingdom. Informed consent was taken from all the students who took part in this study.

An anonymous online questionnaire, designed by the author was distributed to medical students who came for their clinical rotations at the hospital. The questionnaire aimed to identify the primary areas where students felt that they needed help and that aided in the creation of a new teaching program that was introduced in April 2023, to bridge those gaps. The progress of the teaching program was gauged by a second survey conducted after six months, in October 2023 to ensure continuous improvement and evolvement of the teaching program. Participation in the study was anonymous and voluntary, and fully informed consent was taken before participation. No incentives or payments were given for participating in the study and participants were informed that the data would be utilized solely for educational purposes. The data were analyzed using a Microsoft Excel (Microsoft Corp., Redmond, WA) spreadsheet. Categorical data were assessed using measures of central tendency, frequencies, and percentages.

Inclusion criteria

Third and fourth-year medical students who had just started their clinical rotations and who provided informed consent.

Exclusion criteria

Medical students from junior years, i.e., first and second year who had not started their clinical rotations yet or students who did not give consent.

Questionnaire tool

A Microsoft form-based questionnaire was designed for this study. The standard used for designing the questionnaire was the “Medical Licensing Assessment (MLA)” content map. The MLA content map sets out the core knowledge, skills, and behaviors needed for practice in the United Kingdom. The form consisted of two parts. In terms of clinical education, the first section of the form sought to investigate the areas in which students felt they required assistance. This was done by asking specific questions related to students’ performance and participation in clinics and wards such as their preparation for ward rounds, their ability to take patient history in wards confidently, and being able to discuss patient care plans and management with the rest of the team. Further questions aimed to explore how much students learned about clinical procedures, teamwork, and bedside examinations in wards and how confident they were in using their theoretical knowledge in practical situations. Each question was given a score ranging from 1 (not confident/satisfied at all) to 10 (very confident/satisfied) with gradual variations.

The second part of the questionnaire focused on the teaching program and its improvement by asking questions such as how satisfied they were with the teaching sessions delivered and what improvements they suggested to this program so that the education program could be dynamic and well suited to the students’ needs.

Ethical considerations

The study adhered to the World Medical Association's Helsinki Declaration (1964), most recently revised in 2008. All participants were requested to provide informed consent for the study before completing the questionnaire, assuring that all data would be processed anonymously. Participants did not provide any personal information, no identifying information was collected, and all answers were kept confidential. The study received a full Ethical Review Committee (ERC) exemption at Basildon University Hospital, which permitted the survey to be conducted with anonymous participation from the students.

## Results

**Figure 1:**
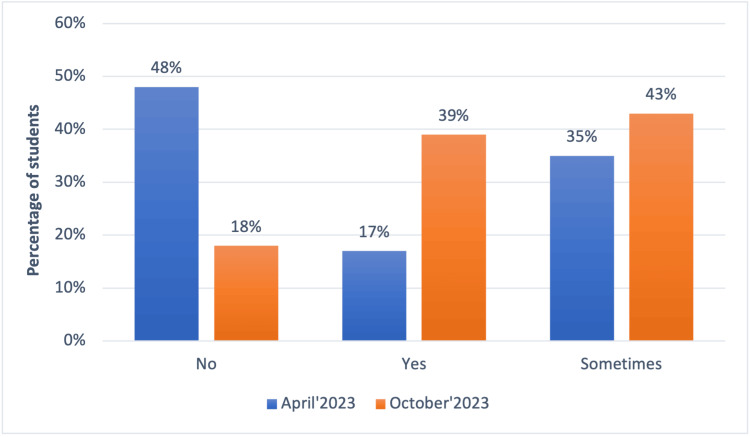
Do you feel adequately prepared for ward rounds? The graph represents percentage of students in April and October 2023, respectively.

The students gave the program an overwhelmingly positive response, feeling that it was aptly suited to their needs. Twenty-three students took part in this study and the results showed that 39% of the students felt adequately prepared for ward rounds in October 2023 in contrast to only 17% in April 2023 (Figure 1).

**Figure 2:**
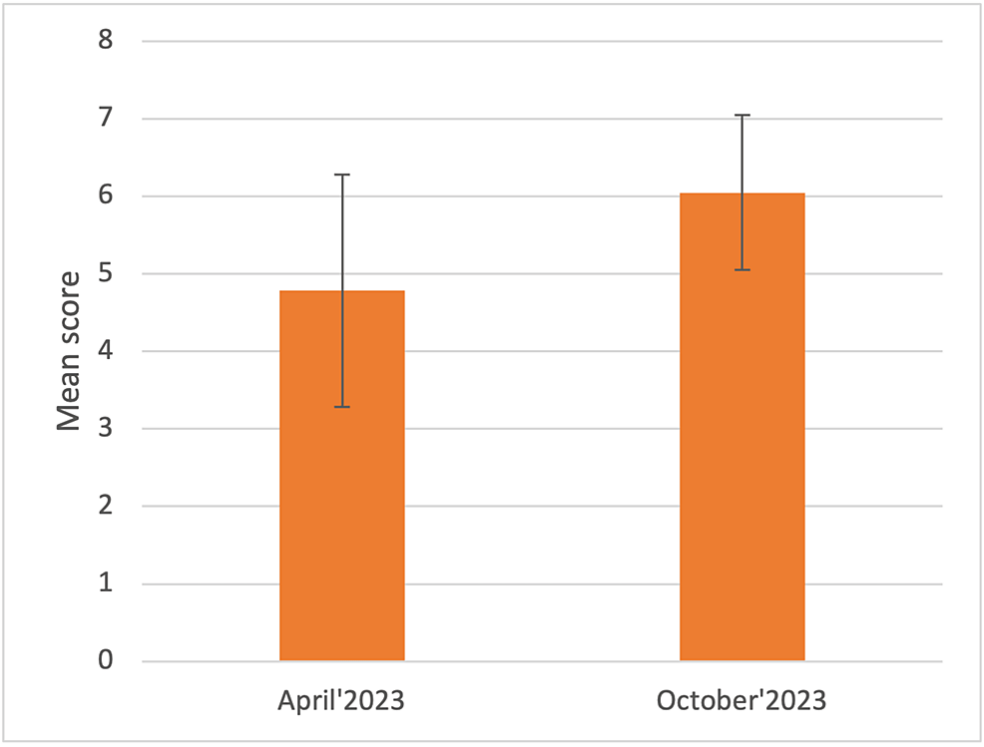
On a scale of 1-10, how confident do you feel in your ability to take patient history in wards? The graph represents the Mean score ± 1 Standard Deviation in April and October 2023, respectively.

Next, a question about the students' confidence in their abilities to obtain patient histories in wards was posed and the data collected showed that on a scale of 1 to 10, the mean score was 4.78 ± 1.5 in April 2023, which improved (p<0.002) to 6.04 ± 0.99 in October 2023 (Figure 2).

**Figure 3:**
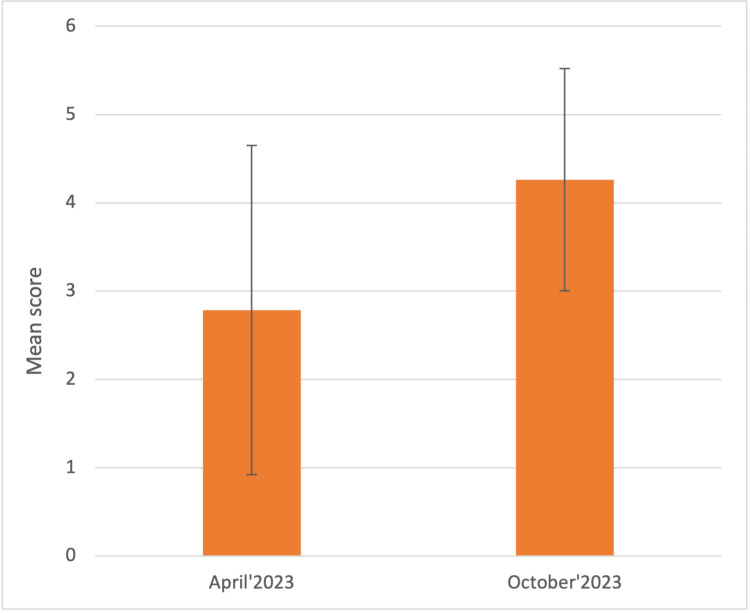
On a scale of 1-10, how confident are you in discussing patient care plans and management with the rest of the team? The graph represents the Mean score ± 1 Standard Deviation in April and October 2023, respectively.

The next question sought to assess students’ confidence in discussing patient care plans and management with the rest of the team and the results demonstrated that students were the shyest in this category, with a mean score of 2.78 ± 1.86 (on a scale of 1 to 10) in April 2023, which significantly improved (p<0.003) to 4.26 ± 1.25 in October 2023 (Figure 3).

**Figure 4:**
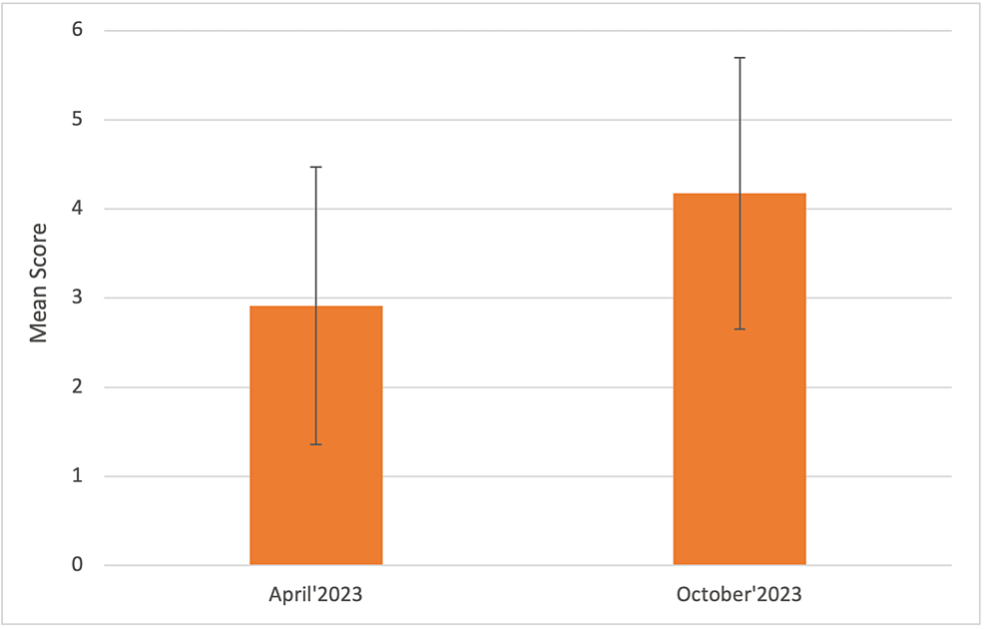
On a scale of 1-10, how confident are you in performing clinical procedures in wards? The graph represents the Mean score ± 1 Standard Deviation in April and October 2023, respectively.

Then we tried to investigate how confident students felt in performing clinical procedures in wards and the data showed that on a scale of 1 to 10, the mean score was 2.91 ± 1.55 in April 2023, which improved to 4.17 ± 1.52 in October 2023 (Figure 4).

The next question aimed to find out how confident students felt in performing bedside examination in wards and the feedback showed that in April 2023, on a scale of 1 to 10, 47.82% of the students scored between 1 and 3 for this question, 39.13% scored between 4 and 6 while only 13.05% scored between 7 and 10. In October 2023, the results improved to 13.04% of the students scoring between 1 and 3 for this question, while 56.52% scored between 4 and 6 and 30.43% scored between 7 and 10 (Table 1).

**Table 1:** On a scale of 1-10, how confident are you in performing bedside examination in wards?

Scale	Percentage of students (April’2023)	Percentage of students (October’2023)
1 - 3	47.82%	13.04%
4 - 6	39.13%	56.52%
7 - 10	13.05%	30.43%

**Figure 5:**
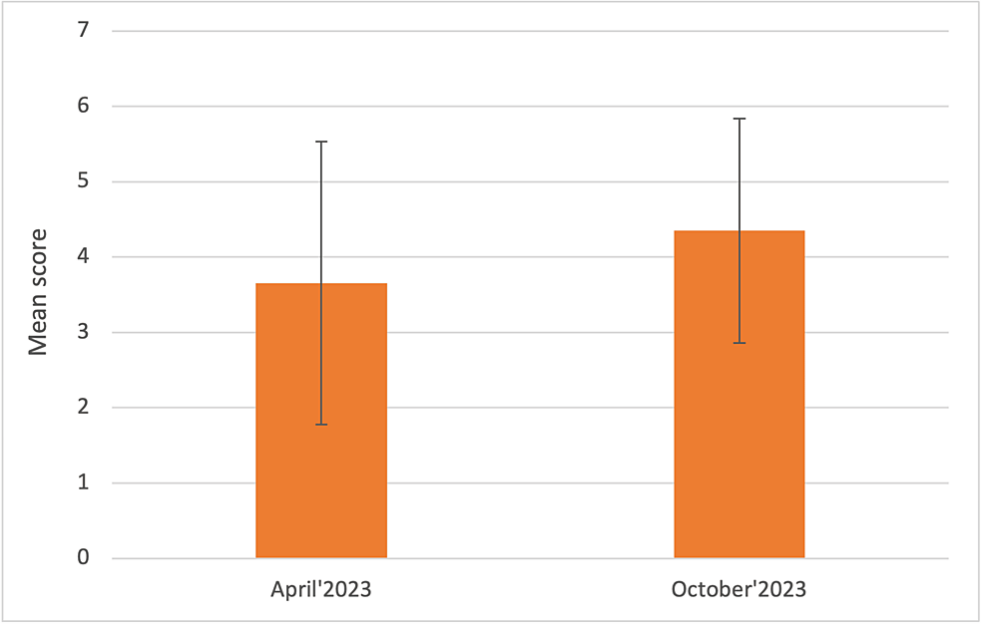
On a scale of 1-10, how confident do you feel in your ability to use your theoretical knowledge in practical situations? The graph represents the Mean score ± 1 Standard Deviation in April and October 2023, respectively.

Then we wanted to find out how confident the students felt in using their theoretical knowledge in practical situations and the answers showed that on a scale of 1 to 10, the mean score was 3.65 ± 1.87 in April 2023, which significantly improved to 4.35 ± 1.49 in October 2023 (Figure 5).

**Figure 6:**
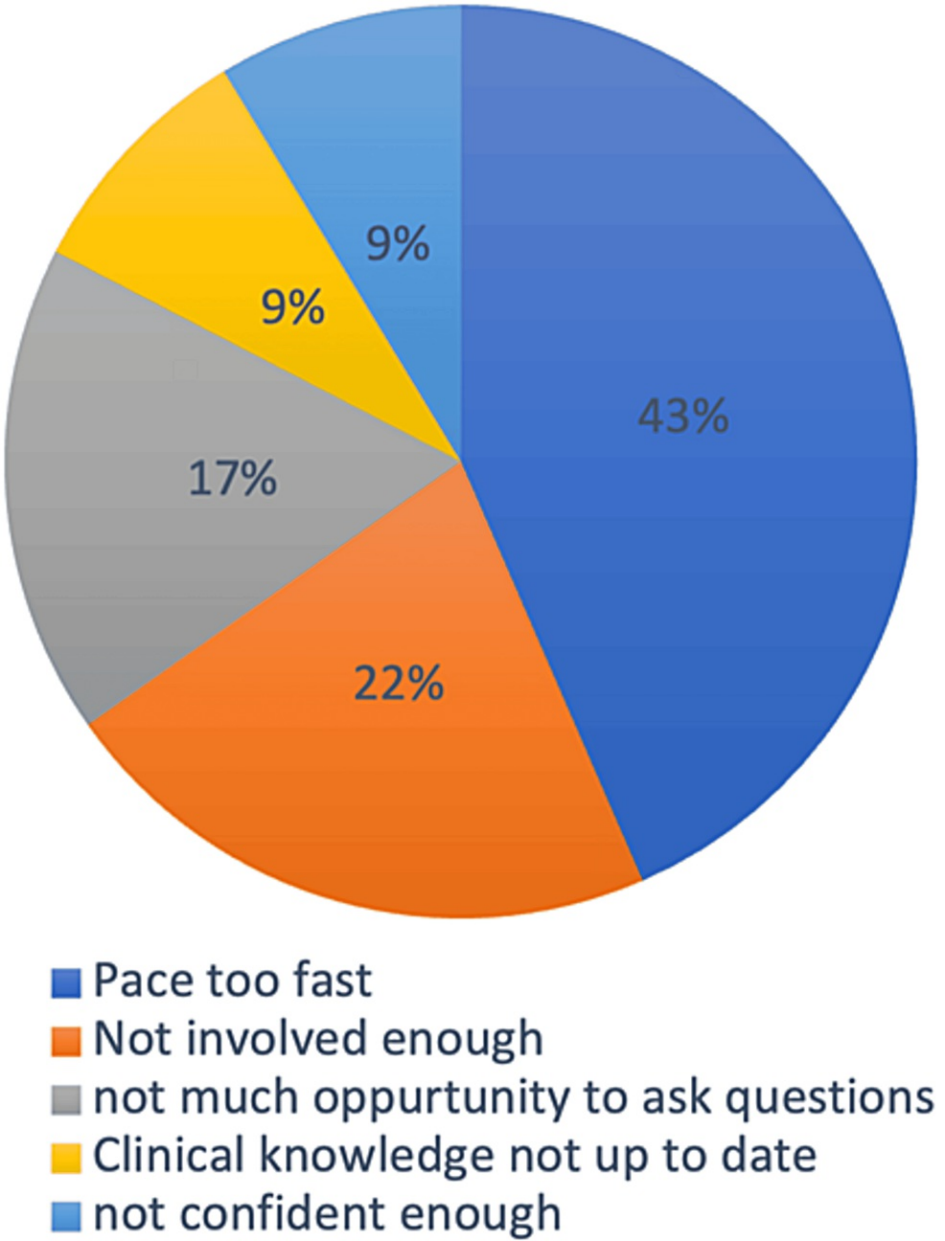
What challenges have you faced in wards and clinics? The values represent percentages.

The next part of the study aimed to identify the challenges students faced in wards and clinics and the responses collected showed that 43% students felt that the pace of wards rounds and clinical activities was too fast for them to catch up, 22% felt that they were not involved enough, 17% responded that there was not much opportunity to ask questions, 9% felt that their clinical knowledge was not up to date and another 9% responded that they did not feel confident enough (Figure 6).

**Figure 7:**
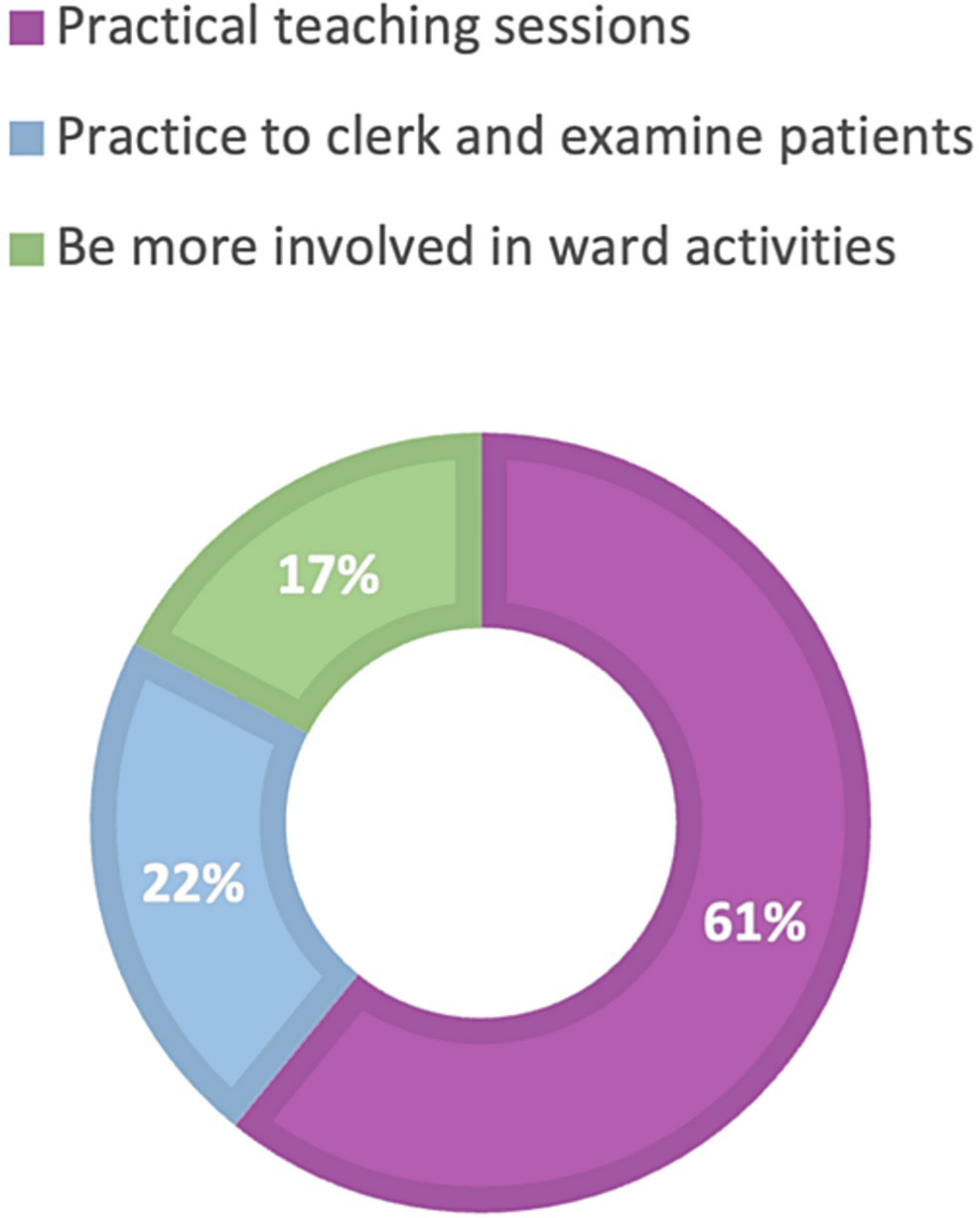
What do you think would help you feel more confident in wards and clinical situations? The values represent percentages.

Then we tried to explore what the students thought would make them more confident in wards and clinical situations and the responses collected showed that 61% of the students felt that they would benefit from more practical teaching sessions, 22% responded that they needed more practice to clerk and examine patients and 17% thought that they should be more involved in ward activities (Figure 7).

**Figure 8:**
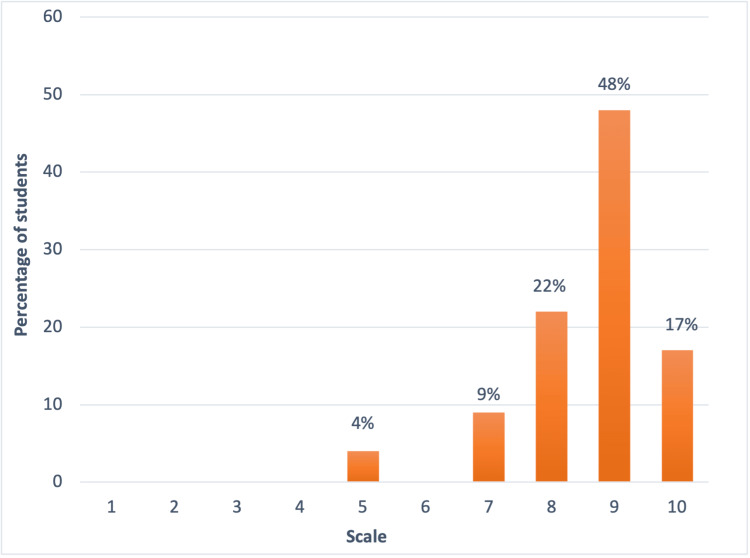
On a scale of 1-10, how satisfied are you with the quality of teaching sessions provided? The values represent percentages.

The second part of the questionnaire focused on the teaching program and its progress. In order to evaluate that, the first question we asked was how satisfied the students felt with the quality of teaching sessions provided and the results showed that on a scale of 1 to 10, 4% of the students gave it a score of 5, 9% scored 7, 22% scored 8, 48% scored 9 and 22% gave it a score of 10 (Figure 8).

**Figure 9:**
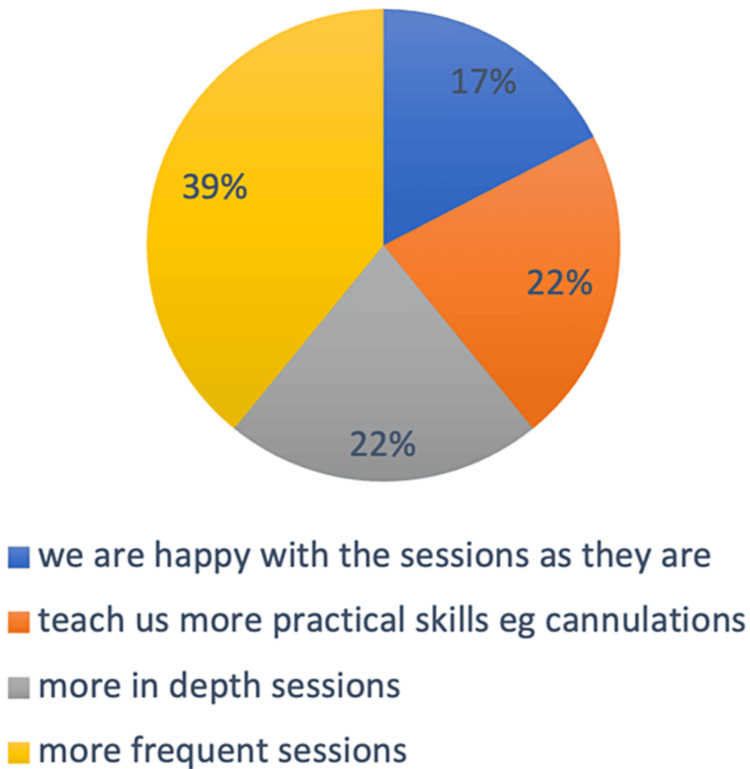
What improvements do you suggest to this teaching program? The values represent percentages.

The next question aimed to identify potential areas of improvement in the teaching program and the students were asked what improvements they suggested to this teaching program and 39% of the students voted that they needed more frequent sessions while 22% felt that the sessions should be more in depth. Another 22% voted that they needed more practice with skills such as cannulations and 17% said that they were perfectly happy with the teaching program as it is (Figure 9).

## Discussion

With the development of technology, healthcare delivery, and medical understanding, it is critical that future physicians possess the skills and information required to ensure the best possible care for their patients. The role of medicine in the diagnosis and treatment of disease, changes in practice patterns, innovations, and advances in medical science must all be incorporated into medical education to adequately prepare aspiring physicians. In order to get the best learning outcomes, healthcare education must use a variety of approaches and strategies to keep students interested and involved. This paper delves into a range of techniques that we tried to achieve this.

We used two questionnaire-based surveys, the first in April 2023, which was conducted before the development of our teaching program to find out the areas where students felt they needed the most assistance, and a second one in October 2023 to compare the pre- and post-teaching results. As demonstrated above, the students felt that teaching should be more practical and clinical-oriented with hands-on practice, rather than theoretical. To achieve this, various techniques were employed, including simulation-based learning.

Simulation-based learning is amongst the best techniques for achieving maximum learning outcomes. Health workers can benefit from simulation-based learning by developing their knowledge, abilities, and attitudes while protecting patients from unnecessary risks [6]. It takes both science and art to practice medicine, and confidence and skills can be developed via repeated exposure to challenging situations, and this can be accomplished by simulation-based learning in a very safe and secure way.

The term “simulation” refers to the artificial representation of a real-world process to achieve educational goals through experiential learning, or it can be used to describe a scenario in which a specific set of conditions is created artificially to study or experience something that is possible in real life [7]. Any educational activity that uses simulative aids to imitate actual clinical situations is considered simulation-based medical education. The use of simulation tools can be used in place of actual patients. Trainers do not have to worry about upsetting patients when they make mistakes and learn from them [8]. Consequently, using simulation techniques in the classroom is one of the best ways to increase students' self-confidence and promote active engagement. Medical errors contribute to the cost of medical care throughout the world and continuous practice through simulation-based learning has been found to be extremely effective at mitigating some of these costs. We attempted to use high-fidelity mannequins to deliver simulation-based learning, and this significantly increased student participation and engagement. Clinical scenarios were thoroughly practiced in detail on mannequins and this significantly improved students' history-taking, examination, and patient management skills as demonstrated by the improvements in mean scores in all these categories.

The purpose of introducing a new teaching program was to foster maximum student participation, which identified the gaps in clinical learning and tried to fill them. An effective teaching program should be diverse, flexible, and based on the idea of “multiple intelligences.” The theory of multiple intelligences was initially proposed in 1983 by Howard Gardner, a developmental psychologist at Harvard. According to this idea, human intelligence can be classified into the following modalities: interpersonal, intrapersonal, logical-mathematical, musical-rhythmic, verbal-linguistic, visual-spatial, naturalistic, bodily-kinesthetic, and existential. As opposed to other theories of learning capacities (such as the idea of a single IQ), the theory of multiple intelligences postulates that individuals learn in a multitude of diverse ways. Gardner states, “I think that over millions of years, the brain has evolved to be responsive to different kinds of content in the world.” [9]. Gardner says that you should present learning materials in a variety of ways, regardless of the subject you teach “the arts, the sciences, history, or maths.”

The multiple intelligences identified by this theory are interpersonal which is the ability to recognize and react suitably to the feelings, intentions, and motives of others. Intrapersonal is the capacity to be self-aware and sensitive to one's own emotions, values, beliefs, and thought processes. Logical-mathematical which is the aptitude for understanding numerical and logical patterns; musical-rhythmic which is the potential for producing and appreciating tone, rhythm, and pitch. Verbal-linguistic which is a strong command of language and awareness of the rhythms, sounds, and meanings of words; visual-spatial which is the ability to think in images and pictures. Naturalistic intelligence is the ability to recognize and categorize plants, animals, and other objects in nature, and bodily-kinesthetic intelligence is the ability to control one’s body movements and to handle objects skillfully [10]. In an effort to deliver optimal education, we structured our classes to stimulate various intelligences, and this significantly enhanced student engagement. We presented a wide range of medical and surgical topics to the students, alternating between several teaching styles in order to maximize learning by stimulating different intelligences. The result of increased student participation was that many students felt more prepared for wards and clinics in October 2023, as compared to April 2023.

Another technique we tried in our teaching sessions was the “Flipped Classroom.” Flipped classrooms are a sort of blended learning method that tries to improve student engagement and learning by having students complete readings and view presentations at home and working on real-world problem-solving during class [11]. This allows time for more active and interactive learning. Under a flipped classroom model, students take charge of their own education and set their own pace for learning while the teacher assists them rather than just imparting knowledge [12]. We attempted this strategy and created workbooks that were accessible to students prior to lectures, giving them time for introspection and contemplation. This really aided in promoting active discussion during class periods. This was accompanied by graded quizzes during each lecture which encouraged brainstorming and a problem-solving approach. We were able to increase student interaction and make the lecture sessions more engaging and interesting thanks to the idea of the flipped classroom. The flipped classroom promoted analytical thinking, and this greatly helped the students to apply their theoretical knowledge to practical situations, as highlighted by the improvement in mean scores for this question in October 2023 as compared to April 2023.

The teaching program we designed aimed to encourage maximum student participation without making anyone feel targeted or too pressured. This was accomplished using the “Learning zone model.” This model was developed by the educator Tom Senninger and is based on Lev Vygotsky’s “Zone of proximal development (ZPD).” It divides the experience of learning into three main zones: the comfort zone, the learning zone (or growth zone), and the panic zone [13]. It illustrates how we need challenges in order to learn effectively. Achieving the ideal balance is crucial, as too little pressure would probably prevent us from venturing outside of our comfort zone, while too much pressure will cause us to become anxious and feel overpowered. Learning is constrained in both situations. The “sweet spot” that is the Learning Zone is what we should instead strive for.

This concept is based on the ZPD by Lev Vygotsky. The ZPD is defined as the area between what a learner can accomplish on their own and what they can accomplish under the supervision of an adult or in cooperation with more experienced peers [14]. This is the area where optimum learning is achieved because ZPD bridges the gap between current and potential ability. What a learner does with help today, will be able to do independently tomorrow [15]. We introduced these two models into our teaching sessions where we attempted to challenge students to step outside of their comfort zones without making them feel overly nervous or panicked. We structured the sessions so that the teacher served primarily as a facilitator, offering support and direction when needed, but the students completed the majority of the problem-solving and critical-thinking tasks on their own. This strategy was quite helpful in increasing student interest and participation. We attempted to find out about the challenges students faced in wards and clinics and the results showed that many students felt that either the pace of activities was too fast for them, or they were not involved enough. The concept of ZPD really helped in tackling this issue where students were allowed to brainstorm at their own pace, the instructor was just the facilitator and everybody was encouraged to participate, with the students receiving guidance and help from the teacher as and when needed.

Additionally, our teaching program included graded quizzes to gauge students' learning progress and offer a platform for additional learning. Before every lesson, we would give the students a quiz to keep them motivated and involved and to find out what prior information they needed to brush up on. We covered both medical and surgical topics in our lessons, and we also presented the idea of “medical champion” and “surgical champion,” which were merits that would be given to the students who performed the best on these quizzes throughout their rotation with us. The concept of fostering a little healthy competition was a big help in raising the students' spirits and excitement. Several studies have demonstrated that when students engage in some healthy competition, they perform better and are more enthusiastic [16,17].

Constructive criticism has been shown to be a powerful tool for making anything better [18] therefore, to guarantee the ongoing development and evolution of our teaching program, we consistently gathered feedback from our students. The students provided us with suggestions on what they thought should be improved in the lessons, and we also provided the students with feedback so they would know where they needed to put in a little more effort. It is very important that feedback communicated to the learner should be intended to modify his or her thinking or behavior to improve learning [19]. The method for giving students feedback was thoughtfully developed. Our goal was to offer constructive criticism without discouraging anyone, thus we used the “Star star wish” approach as our example. In this technique, the teacher identifies two areas where students excelled (stars) and one area where they can improve (wish). So, by first encouraging and appreciating students on what they did well and then telling them about potential areas for improvement, this technique ensured that feedback was constructive and encouraged growth.

The success of our teaching program was largely attributed to the combination of all these strategies. Students thoroughly enjoyed the sessions and expressed great satisfaction with the program. We asked the students to score the teaching program on a scale of 1 to 10 (1 being the lowest and 10 being the highest) and the majority of the students (48%) gave it a score of 9.

Our future plans include making our teaching sessions a bit more digitally advanced. We found that one area that needed improvement was the ability for students to ask questions anonymously. This is because some students are hesitant and do not ask many questions. We plan to do this by using an app called “mentimeter’’ which can allow students to ask questions anonymously and as many times as they want without any reluctance. Additionally, we want to give students the option to contribute and engage in sessions in an anonymous manner. To this end, we intend to employ the “padlet” app, which will allow students to freely discuss, brainstorm and present their ideas to the group without worrying that they would sound foolish.

There were certain limitations to our study. Firstly, our sample size was small including only medical students who came for their clinical rotations to the hospital, so the findings of the study cannot be generalized. Therefore, to gain more insights into the effectiveness of different teaching techniques for medical students, future studies with larger sample sizes and more diverse student populations are recommended. Secondly, we employed an online survey form, which may have contributed to a lower response rate, possibly due to volunteer bias. Since there is no in-person interaction between the survey administrator and the respondent to address queries regarding the questionnaire, our data collection approach may potentially be vulnerable to other forms of bias. We hope that our study will serve as a springboard for more research on this topic and on enhancing the delivery of medical education.

## Conclusions

Many medical students expressed satisfaction with the teaching program and called for more regular sessions. The results of this study demonstrated that instructors must use a variety of strategies and approaches to promote greater student involvement and effective participation. The study proved that a multi-modal teaching program that engages various bits of intelligence and evolves according to the students' needs is indeed effective at improving students' clinical, practical, and analytical skills and can greatly help in enhancing the delivery and reception of healthcare education.

Simulation is an excellent technique that can be used in healthcare education without posing any risks to patients. The more the students practice through simulation techniques, the more confident they will feel in real clinical scenarios. Other techniques such as flipped classrooms have been shown to be very effective too, as they take away the burden of long readings and allow more time for active discussions and problem-solving through brainstorming. Enhancing students' analytical abilities and encouraging critical thinking should be the main goals of the education program. Students learn best when they are assisted and actively involved, as demonstrated by Lev Vygotsky's ZPD. So, a good teaching program should be diverse, and interactive and encourage a bit of healthy competition amongst students in order to keep them engaged and interested.
